# Biotechnical Multiscale Engineering of Scaffolds for Stem Cell and Organoid Research

**DOI:** 10.1002/smll.202504070

**Published:** 2025-10-30

**Authors:** Merle‐Johanna Küstner, Christian Marx, Peter beim Graben, Serafim Rodrigues, Jörg Hampl, Frank Weise, Michael Gebinoga, Leon Kaysan, Maren Klett, Dana Brauer, Gregor Schlingloff, Doris Heinrich, Insa Sigrid Schroeder, Andreas Schober

**Affiliations:** ^1^ Ilmenau University of Technology Ehrenbergstraße 29 98693 Ilmenau Germany; ^2^ GSI Helmholtzzentrum für Schwerionenforschung GmbH Planckstraße 1 64291 Darmstadt Germany; ^3^ Bernstein Center for Computational Neuroscience Berlin Humboldt‐Universität zu Berlin Bernstein Center for Computational Neuroscience Berlin Unter den Linden 6 10099 Berlin Germany; ^4^ BCAM Basque Center for Applied Mathematics Alameda de Mazarredo 14 Bizkaia Bilbao 48009 Spain; ^5^ Ikerbasque ‐ the Basque Science Foundation for Science Plaza Euskadi 5, Bizkaia Bilbao 48009 Spain

**Keywords:** biophysical observation models, biotechnical multiscale engineering, brain organoids, hematopoietic stemcells, micro/nanotechnology, orgamats, scaffolds

## Abstract

The publication describes complex support structures and scaffolds for stem cells and organoids. Consideration of geometric and structural parameters has an influence on stem cell development and organogenesis comparable to that of molecular genetics and biochemical parameters. Two essential representatives are discussed here in more detail: hematopoietic stem cells (HSCs) and brain organoids. Due to their ability to fully regenerate the blood system, HSCs are used for stem cell transplantations. Therefore, efficient approaches to create an artificial but close‐to‐nature stem cell niche in vitro for amplification of HSCs are highly desirable. Apart from biochemical and biological factors, geometrical and biomechanical parameters are important. Biotechnological multiscale engineering is able to mimic the HSC niche, improving their amplification. Another highly dynamic process underlying hierarchic orders is organogenesis. Entire organs develop from individual cells that are spatially arranged in precise patterns and exposed to chemical, mechanical, and structural stimuli. The significance of the different scales during their development is explained using human brain organoids. Here, geometrically suitable structures improve biochemical differentiation protocols. Such technical hybrid systems can foster research of a rather inaccessible organ and possibly serve as a platform for more energy‐efficient computing devices, such as organoid automata, hence, *orgamats*.

## Graphical abstract

Biotechnical multiscale engineering consideration of geometric and structural parameters, which is called biotechnical multiscale engineering, has an influence on stem cell development and organogenesis comparable to that of molecular genetics and biochemical parameters. Two essential representatives are discussed here in more detail: hematopoietic stem cells (HSC) and brain organoids.

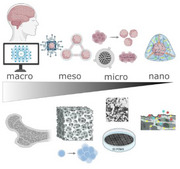

## 1 Introduction

In reference to the famous quote by Richard P. Feynman, “What I cannot create, I do not understand”,^[^ ^1^ ^]^ this would mean, in the case of life, that we would have to create living beings from scratch to make them real. Even now, despite the big success of modern synthetic biology, stem cell research, and nano/microtechnology, this aim seems to be far beyond the realm of possibility. Apart from the lack of understanding of the creation of living beings from the beginning, the number of parameters, the feedback of evolving, self‐organizing biosystems on their own constraints, makes it challenging to generate conditions or state spaces that allow the creation or even the prediction of such systems from scratch. Perhaps it is better to follow another quote from the famous Austrian author Robert Musil “*Wenn es einen Wirklichkeitssinn gibt, muß es auch einen Möglichkeitssinn geben*”; English: “If there is a sense of reality, there must also be a sense of possibility.” Following this quotation, the implication for our case is in developmental biology, the potential space of possible forms could be explored with the help of stem cell‐based embryo models. Sufficient complexity must be created in the model systems. In addition to a necessary genetic component, size, order, and structure also play an important role. For example, the liver is an organ at the centimeter scale, partitioned into liver lobules, typically a few millimeters in diameter. The liver lobules, in turn, are arranged in sinusoidal structures in the micrometer range, and the sinusoids themselves are composed of nanoscale elements and structures.

It should therefore be possible to achieve potential pathways in morphospace not only by genetic manipulation, but also by altering the boundary conditions, thus revealing potentially hidden morphogenetic possibilities, for example. The relationship between gene expression and cell and tissue mechanics, self‐organization, the role of the threedimensional (3D) extracellular matrix (ECM), and extraembryonic tissues are some examples to be mentioned.^[^ ^2^ ^]^ In this research design, concrete biological questions can be implemented as in vitro models in hybrid systems consisting of biological and technological components, or even abstract questions about the evolution of complex systems can be subjected to experimental investigation as subdivided aspects in corresponding experimental systems. The great attraction lies in the fact that for each level of description, an in vitro model can be realized as a biological‐technical hybrid system in a laboratory setup (or, in the sense of biotechnological multiscale engineering, all levels of description can be adequately captured in such a setup).^[^ ^3^, ^4^ ^]^ Thus, the theoretical modeling of the various levels of description and their potential transitions can also be captured in an experimental setup. Modeling at such levels of description helps to understand the findings of neurophysiological research as one example in a reductionist, but not trivial, way. Compartmentalization, for example, allows for the study of different cell and functional types and their interactions via defined channels. Furthermore, such systems can also be used to investigate the conditions for specialization and hierarchization of information and function.

As an example, an aspect of our research activities and that of many other groups concerns the improved propagation of stem cells for therapeutic applications (e.g., HSCs for transplantation) as well as improved in vitro differentiation of stem or progenitor cells mimicking organogenesis of tissues of interest (e.g., the otherwise inaccessible human brain tissue represented by brain organoids) and their integration in hybrid systems. In the case of HSCs, this comprises the generation of an artificial but close‐to‐nature stem cell niche. Regarding the generation of human brain‐like tissue, this includes the assembly of brain organoids and assembloids, generated by fusion of two or more organoids of different entities; the directed growth of neuronal palisades on plastic supports with integrated electrodes; the integration of the plastic support‐neuron bundle composites into ceramic electrode structures; and the subsequent measurements of pointwise dendritic field potentials (DFP), local field potentials (LFP) and total electroencephalographic (EEG) field potentials.^[^ ^5^, ^6^, ^7^ ^]^ Our goal is a better understanding of the structure and function of the cortical column.^[^ ^8^, ^9^, ^10^, ^11^, ^12^, ^13^ ^]^ Such brain organoids and functional assembloids would also be mandatory for testing and improving biophysical observation models in computational neuroscience.^[^ ^5^, ^6^, ^7^ ^]^ Moreover, recorded and appropriately processed field potentials could be fed back to neural populations in a closed‐loop arrangement to train them for the fulfillment of computational tasks, as it has been achieved so far with twodimensional (2D) neuronal cultures in the animate paradigm.^[^ ^14^, ^15^ ^]^ Thus, 3D organoids and assembloids could biologically implement neural automata,^[^ ^16^, ^17^ ^]^ or *orgamats*, as sustainable future computing devices.

Therefore, nanobiosystem research is dedicated partly to the examination and reproduction of biological systems from the aspect of biotechnical multiscale engineering (BME).^[^ ^3^, ^18^ ^]^ Hence, we think of a systems‐biology perspective assuming macroscopic biological systems also represent a synthesis of functional nano‐ and microelements. For the systematic approach and the associated post‐modeling, it must therefore be taken into account that both the smallest functional elements and the next higher units are necessary for the function of biological systems.^[^ ^3^ ^]^ This leads us to either use nano‐ and microtechnology tools to examine cells and tissues, or to guide cellular behavior with structured functional materials. In this perspective, we present and discuss some examples and concepts related to hybrid systems. Here we propose 1) the technical construction of the HSC niche, 2) the development of human brain organoids by the BME approach, and their potential application in a biological‐technical hybrid system, and conclude 3) with a short resume.

## 2 The Technical Construction of the Hematopoietic Stem Cells (HSC) Niche

HSCs have the ability to self‐renew, the capacity for unlimited proliferation, and the potential to differentiate into all types of blood cells and thus to regenerate the blood system.^[^ ^19^, ^20^ ^]^ Since mature blood cells have a limited lifespan, they must be continuously reproduced by differentiation from HSCs in order to maintain the hematopoietic system.^[^ ^19^, ^20^ ^]^ Because of these abilities, HSCs are also used for transplantation (HSC transplantation, HSCT), a procedure that treats a variety of diseases, including autoimmune diseases, blood disorders, and certain cancers that cannot be treated otherwise.^[^ ^21^ ^]^ Patients benefit from high numbers of transplanted HSCs; however, despite constant development, there are often not enough stem cells available in the donor material, which significantly reduces the success of HSCTs.^[^ ^20^, ^22^, ^23^ ^]^ Therefore, efficient approaches to create an artificial but close‐to‐nature stem cell niche in vitro for amplification of HSCs are highly desirable. An overview of the hematopoietic stem cell niche, its structure and components, is given in recent review articles.^[^ ^24^, ^25^, ^26^ ^]^

**Figure 1:**
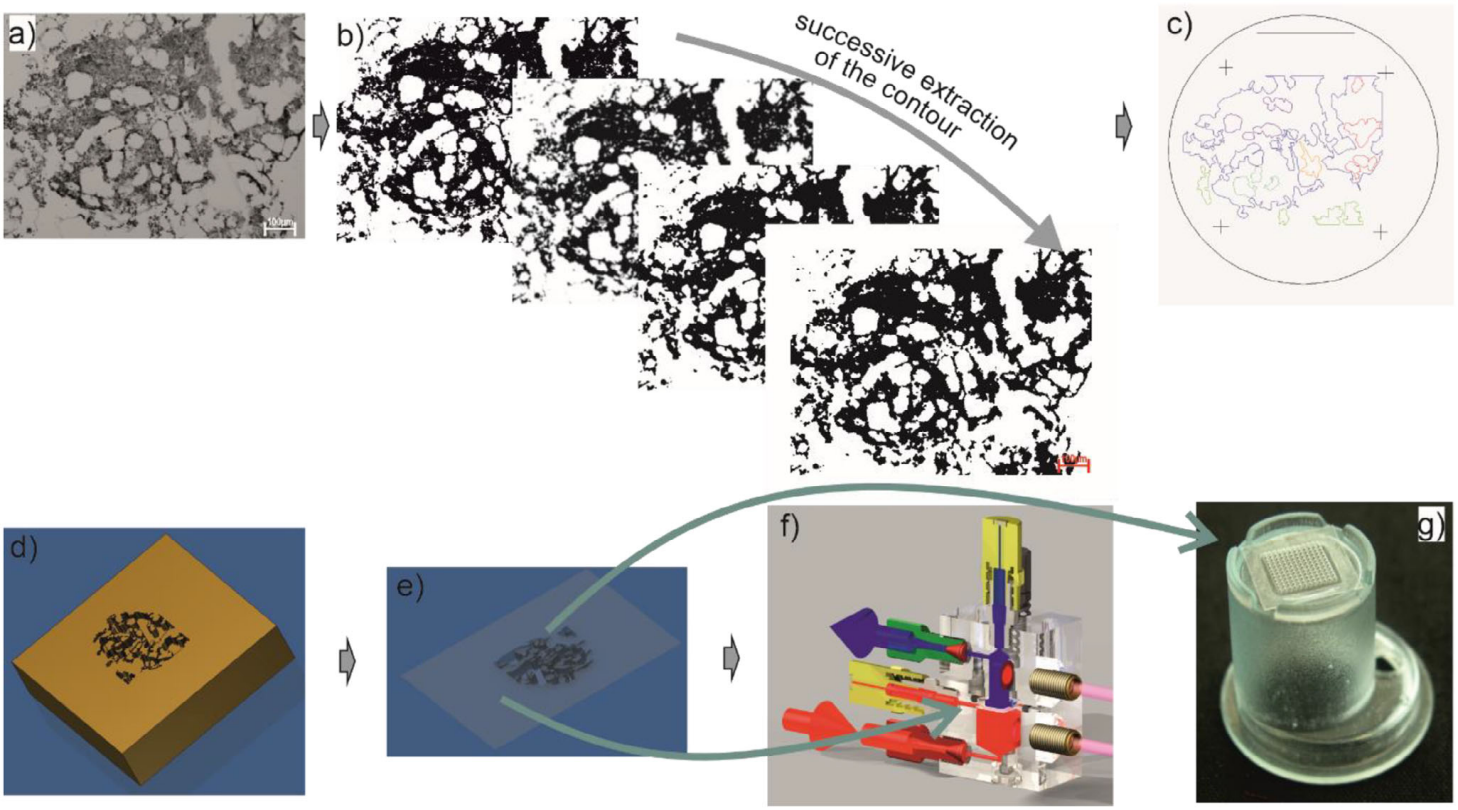
Reproduction of the human hematopoietic stem cell (HSC) niche onto scaffolds for the in vitro amplification of HSCs.^[^ ^43^ ^]^ The nano‐scale environment of the HSC niche is transferred in a step‐wise process onto macro‐scale polymeric structures for in vitro cell cultivation. a) An image of the bone marrow (BM) of a human long bone cross‐section biopsy was taken and used for image processing to design the scaffold. b) The contour was extracted using MATLAB and “Mathworks File Exchange”. Images were converted to grayscale to create a binary image using a threshold filter (Niblack) and were vectorized by CorelDraw. c) Data were further processed with AutoCAD. The original image (1 mm x 1 mm) was patterned and aligned into a 2D array fitting the diameter of one well of a 24‐well plate (1.9 cm^2^), and extracted features were adapted to construct photolithographic masks. d) By micro‐structuring with an ICP process, silicon masters were fabricated and utilized as either casting or stamping molds. In this way, the construction of positive and negative replicates of the image was done (mold, embossing tool). e) Polymer foils were embossed and f) used in a perfused bioreactor system or (g) in a microplate insert system to create 3D PMDS scaffolds. All images copyright TU‐Ilmenau.

The HSC niche is not only defined by biochemical and biological parameters, but also by geometric 3D structures in the bone marrow (BM) and biomechanical parameters.^[^ ^25^, ^27^, ^28^, ^29^, ^30^ ^]^ Investigations into the position of the HSC niche within the BM show, in most cases, that it is located at the interface with the bone. Hereby, other cell types, such as osteoblasts, are interacting with cells of the hematopoietic system, e.g., by the secretion of cytokines.^[^ ^25^, ^26^, ^31^, ^32^ ^]^ In addition, defined helper cells play an important role in the maintenance, proliferation, and differentiation of HSCs in their niche.^[^ ^33^, ^34^ ^]^ The HSC niches are a highly specialized microenvironment and examples of a macroscopic system with micro‐ and nanoscopic structures defining physical and geometric properties and supporting HSC maintenance, proliferation, and differentiation.^[^ ^35^ ^]^ The HSC niche in the BM comprises the generally well‐defined endosteal and (peri)vascular (specifically, arteriolar and sinusoidal) niches, which host HSCs in close proximity to osteoblasts and endothelial cells.^[^ ^36^ ^]^ For example, the human long bone (femur) of an adult is ≈40.3 ± 2.2 cm long with a distal epicondylar width/diameter of 8.1 ± 0.6 cm (macro‐environment)^[^ ^37^ ^]^ harboring the BM and the HSC niche. An area with a distance of 100 µm from the bone is commonly used to define the endosteum. 3D imaging, however, revealed a high density of HSCs (80% of HSCs in the BM niche) in a larger endosteal region from the bone surface of ≈220 µm in the direction of the central vein (micro‐environment).^[^ ^38^ ^]^ Within the BM, different cell types define the environment and the HSC niche. As an example, osteoblasts have a diameter of 20–50 µm,^[^ ^39^ ^]^ whereas human HSCs only have a diameter of ≈8 µm.^[^ ^40^ ^]^ The shape of the endothelial cells varies across the vasculature in the BM, being roughly 50–70 µm long, 10–30 µm wide, and 0.1–10 µm.^[^ ^41^ ^]^ On the smallest scale (nano‐environment), fibers of the extracellular matrix (ECM) like fibronectin (61 nm length and ≈2 nm diameter) or laminin (36–77 nm length and 2–7 nm diameter)^[^ ^42^ ^]^ define the HSC niche. Hence, all of these parameters and scales need to be considered when creating a close‐to‐nature artificial 3D‐culture system for HSCs mimicking their niche (**Figure** **1**).

The replication of the HSC niche, in particular the BM microenvironment, is a field of intensive biomedical research. According to the complex structure of the BM, especially with respect to geometry, composition, and plurality of different materials/cell types, current research is focused on geometric scales and material properties. Researchers are not only trying to mimic the HSC niche microenvironment by adjusting cytokines, cell culture medium conditions, or oxygen pressure^[^ ^43^, ^44^, ^45^ ^]^ but also the stiffness and material properties by designing ECMs.^[^ ^25^, ^28^, ^46^, ^47^, ^48^, ^49^ ^]^ A promising approach to optimize HSC culture conditions has been described in Ref.[50]. The morphology of the BM is achieved by an in vivo system implanted in mice, stimulating the growth of a matrix similar to the one found in the BM. This matrix is then excised from the mice and used in vitro for the HSC expansion and differentiation experiments. Within the scope of this model are bone‐like substrates that can be cleared of residual cells and further used as substrates for proliferation experiments.^[^ ^51^ ^]^

Another possibility to multiply HSCs in vitro while retaining their stem cell properties is the use of synthetic 3D cultivation systems that simulate the stem cell niche in order to create optimal conditions for symmetric division in the sense of self‐renewal.^[^ ^20^, ^43^, ^52^ ^]^ To construct those 3D structures modern micro‐ and nano‐technological methods are applied. In this way, for example, regular, pillar‐like structures in the dimension of micro bone morphologies are realized as cavities for the stem cell^[^ ^53^ ^]^ as well as micro‐structured microcavity arrays ranging from 15 to 40 µm.^[^ ^48^, ^49^, ^54^, ^55^ ^]^ Most of the 3D scaffold‐based experiments described so far are based on cavity systems for HSC culture, similar to the skeletal structure of hematopoietic bones. Such scaffolds are made from agarose, extracellular matrices (Matrigel), synthetic gels, methylcellulose, and solid spongy structures.^[^ ^56^, ^57^ ^]^ Some of these approaches claim to mimic the basic characteristics of natural BM artificially in the lab by creating synthetic sponge‐like polymers. In addition, proteins, which are commonly found within the BM matrix, can be used as anchors for cell contacts coupled to the synthetic material. Other cell types of the HSC niche, such as mesenchymal stem cells, can also be seeded into the scaffolds to improve HSC culture conditions.^[^ ^58^ ^]^

Recently, ceramic scaffolds mimicking BM‐like structures were used in 3D microfluidic bioreactor systems.^[^ ^59^, ^60^ ^]^ In these studies, researchers were modifying the ceramics by engineering the ECM through designed human cell lines or by modifying the chemical properties of the hydrogels, thus even controlling the topography on a nanoscopic scale.^[^ ^61^ ^]^ Some disadvantages of currently available 3D gels, foams, ceramics, or other BM‐like structures are the complicated harvesting of cells after expansion and the lack of transparency, and thus the possibility to observe live cells. To mimic the HSC niche in vitro, we developed artificial 3D structures based on the cross‐section of a human long bone made of polydimethylsiloxane (PDMS) with the aim of solving issues of previous HSC culturing systems.^[^ ^43^ ^]^ For this purpose, the morphology of the human long bone was analyzed and transferred directly to the biocompatible PDMS substrates using a multiscale design of micro‐ and nanotextures and polymer structuring methods (Figure 1).^[^ ^43^, ^52^ ^]^ With this process, we designed structures/scaffolds with the main (microscopic) features of the BM, named 3D PDMS, which are transparent, upward accessible, and can be implemented in a normal 24‐well cell culture plate. So far, we have shown that cells can be easily harvested from these PDMS scaffolds after expansion and that cell growth can be monitored by fluorescence microscopy within these 3D structures.^[^ ^43^, ^52^ ^]^

By optimizing existing cultivation protocols in combination with the 3D PDMS structures, a suitable environment was created to amplify human HSCs during 14 days of in vitro cultivation while maintaining their pluripotency and ability to self‐renew.^[^ ^43^, ^52^ ^]^ We were able to show that the 3D PDMS scaffolds are significantly better suited to effectively multiply HSCs in vitro than 2D (conventional) polystyrene cell culture dishes or non‐structured 2D PDMS surfaces.

Furthermore, we examined the effects of different surface coatings of 3D PDMS scaffolds by flow cytometry analyses.^[^ ^43^, ^52^ ^]^ For example, 3D PDMS structures covered with silicon oxide (SiOn) had an increased surface hydrophilicity and an increase in the overall yield of HSCs, but the subfraction of immature long‐term HSCs was best propagated on uncoated 3D PDMS. Similarly, fluorescence microscopic analyses showed that high numbers of cells within the cavities of the 3D PDMS structures expressed the surface marker CD34, indicative of HSCs. The SiO_n_‐coated 3D PDMS structures appeared to stimulate HSC growth, but at the same time promoted their differentiation into other cell types.^[^ ^43^, ^52^ ^]^

Future research in our group focuses on the examination of induced pluripotent stem (iPS) cell‐derived hematopoietic stem cells (iHSCs) and their behavior in the 3D PDMS structures. Here we continue to follow the idea that the geometry of the BM can be mimicked by geometric measures derived from parameters like equal wetted surface area ratio, wetted surface area to volume ratio, and the porosity of bone structures. These factors might have a direct or indirect influence on the stem cell niche. To be able to test these dependencies, different structures need to be produced that have similar geometric parameters but differ in shape. Ng et al. recently showed that CD34+ iHSCs can be effectively generated from human iPS cells in vitro under standard 2D cell culture conditions.^[^ ^62^ ^]^ After intravenous transplantation into immuno‐deficient mice, these iHSCs produced multilineage BM engraftment in 25–50% of mice, which was comparable to transplantation of umbilical cord blood. Our advanced 3D PDMS cell culture scaffolds in combination with this elegant approach to generate iHSC might help to further improve the numbers of in vitro amplified stem cells and thus, the outcome of transplantation in vivo.

Furthermore, we aim to modify the stiffness and surface parameters of our 3D PDMS scaffolds to investigate their influence on HSC in vitro growth in future studies. Alternative materials, including agarose, Matrigel, or synthetic gels/hydrogels, could be used to replicate 3D BM‐like structures with the existing molds or could also be used as a covering layer on top of 3D PDMS scaffolds to modify the mechanical properties, including stiffness and pore size of the cell culture substrate. This could lead to improved amplification of more naïve HSCs in a softer substrate that is closer to the natural HSC niche.^[^ ^28^, ^35^, ^63^, ^64^ ^]^ Previous studies already showed that the mechanical properties of the culture substrate and the mechanical signaling pathways are important for stem cell in vitro culture and maintenance of their stem cell characteristics.^[^ ^65^, ^66^, ^67^ ^]^ Thus, these parameters need to be considered in future experiments to optimize our 3D PDMS HSC culture scaffolds.

Since 3D PDMS scaffolds are placed at the bottom of normal 24‐well cell culture plates, this offers the attractive possibility to perform co‐culturing experiments using trans‐well inserts. In this way, other cell types from the HSC niche, i.e., osteoblasts or mesenchymal stem cells,^[^ ^68^, ^69^ ^]^ could be co‐cultured in close proximity to the stem cells in the trans‐well inserts, replicating a more natural environment for the in vitro amplification of HSCs without mixing cell populations. The crosstalk between HSCs and other cell types via cell‐cell‐communication is important for their maintenance and natural functions.^[^ ^68^, ^69^ ^]^ By modifying the mechanical properties of 3D PDMS scaffolds and performing co‐culture experiments, we might be able to recreate a more natural environment for HSC in vitro culture to further enhance the amplification of naïve HSCs for applications such as HSCT in the future. Upcoming experiments will be necessary for validation.

In summary, the in vitro cultivation of human HSCs could already be optimized using our 3D PDMS structures; however, further studies are still necessary to improve and establish our 3D PDMS culture system. The application of these in vitro amplified HSCs from 3D PDMS scaffolds in vivo is still pending, but could help to solve a major problem in transplantation medicine in the future.

## 3 Evolution of Cerebral Organoids by the BME Approach and their Potential Application in a Biological‐Technical Hybrid System

The human brain is a complex organ whose processes/uniqueness cannot be adequately reproduced in animal models. Due to the limited availability of primary material and ethical restrictions, patient samples are also not a sufficient alternative. Cerebral organoids serve as a bridge between primary samples and animal models. In combination with iPS cell technology, modeling and knowledge acquisition were achieved in the areas of neurological disorders, psychiatric diseases, neurodegeneration, and evolutionary mechanisms.^[^ ^70^ ^]^ When modeling diseases, however, it must be taken into account that organoids represent a simplified model of the human brain that lacks its natural cell diversity. Especially, non‐ectodermal cells like vascular cells and microglia remain underrepresented in organoids. Additionally, organoids typically lack myelination and organization, resulting in less complex neuronal network activity.^[^ ^71^, ^72^ ^]^ Nevertheless, even as a simplified model, organoids are a valuable tool for representing specific features of human neurodevelopment, especially at early stages^[^ ^73^ ^]^ and broaden our knowledge of the processes during development, disease, and regeneration of the brain. In addition, since the first publication on brain organoids in the last decade, a great research interest has developed in this field. Efforts to increase cell diversity in organoids have shown initial success: Vasculature‐like structures have been introduced into brain organoids^[^ ^74^, ^75^ ^]^ and many approaches are pursued in this respect, ranging from fusing brain organoids with vascular spheroids to culturing brain organoids on microfluidic chips (reviewed in Ref.[76]). Co‐culture systems with microglia have been established.^[^ ^77^ ^]^ Furthermore, protocols for an earlier and more efficient maturation of oligodendrocytes in organoids have been developed.^[^ ^78^ ^]^ In addition to (bio)chemical cues, biotechnological approaches, such as the use of scaffolds and microfluidic systems, can improve the organization and reproducibility of organoids.^[^ ^72^, ^79^, ^80^ ^]^ In this respect, mechanotransducive and spatial factors, as they occur, for example, during the formation of the neural tube, play an important role in the development of the fetal human brain. During neurulation, the neural tube forms from the flat neural plate. Therefore, the neural plate exerts intrinsic forces via the apical constriction of the cells during its formation, and the adjacent tissue exerts extrinsic forces.^[^ ^81^ ^]^ Like in vivo, the mechanical and physical properties of the microenvironment of neuronal organoids have a decisive influence on the development of the organoids.^[^ ^82^ ^]^ The proliferative zones in cerebral organoids show resemblance to the developing fetal cortex.^[^ ^83^ ^]^ However, instead of multiple proliferative zones formed in most organoid approaches, the cortex is developed from one zone originating from the neural tube. Mechanical stimuli are often applied via composition on the surrounding ECM. In most cases, Matrigel is used as the ECM for whole‐brain organoids. As a natural product, the stiffness of this hydrogel may vary, which influences organoid development. In contrast, Isik et al. modified a tyramine‐functionalized hyaluronic acid‐based hydrogel with incorporated peptide amphiphiles carrying a laminin‐derived epitope as an alternative for Matrigel.^[^ ^84^ ^]^ By engineering this hydrogel, the influence of the peptide amphiphile carrying laminin epitopes (nanostructure) alongside varying stiffnesses (mechanical force) of the gel could be tested. Multi‐omics analyses of cerebral organoids grown in engineered hydrogels and in Matrigel revealed that ECM stiffness and nanoscale architecture are key determinants of organoid development. By incorporating the peptide amphiphile with laminin epitopes, a comparable development of the organoids as in Matrigel could be achieved.^[^ ^84^ ^]^ To further improve these hydrogels, additional components of the ECM in the human brain can be incorporated into these gels.

**Figure 2:**
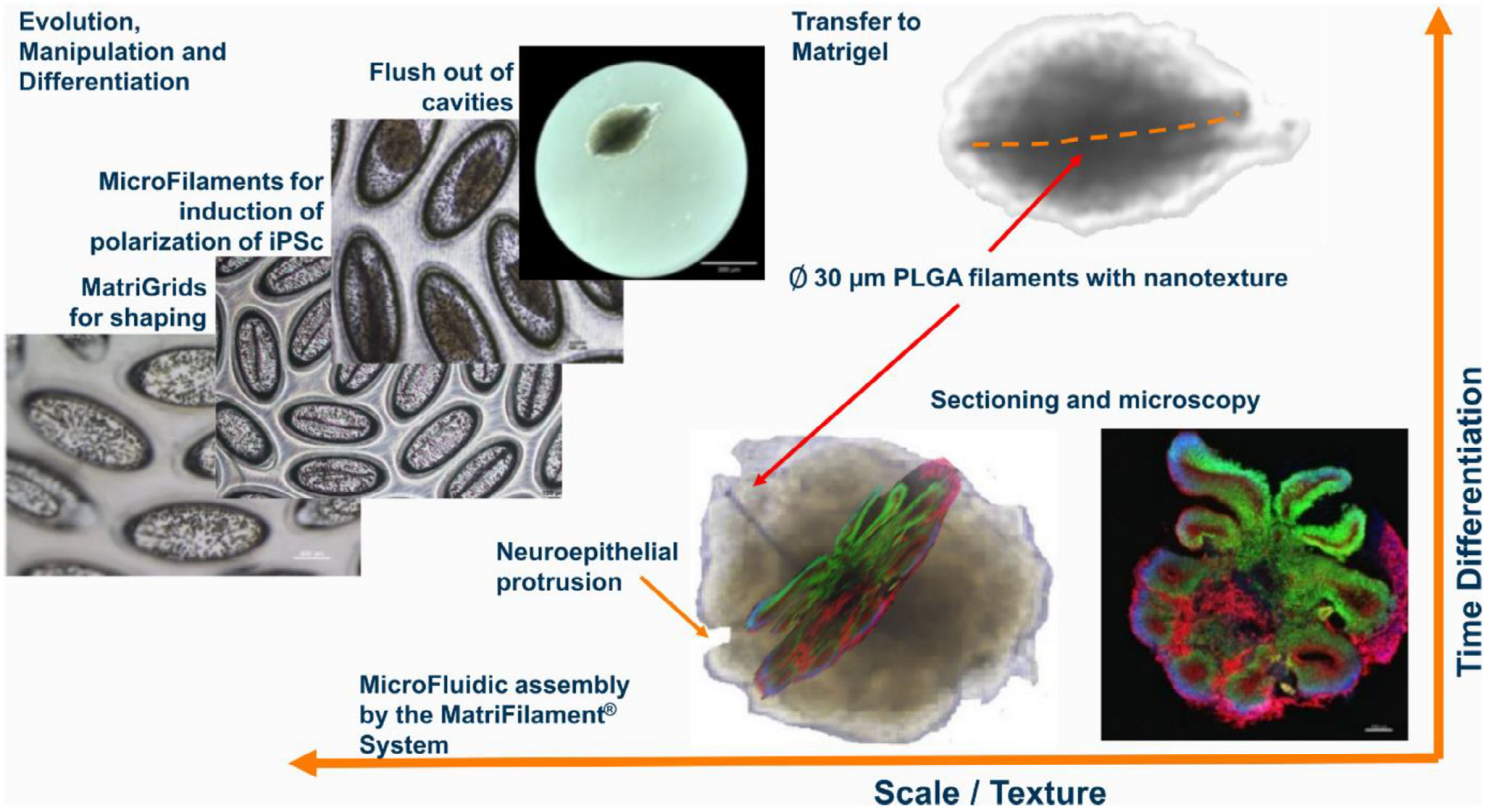
Analogous to early fetal brain development, various factors and scales play decisive roles in the temporal course of the differentiation of brain organoids. The central nervous system initially develops from the thickened neuroectodermal tissue of the neural plate. The spatial arrangement of the cells already plays a decisive role in the successful differentiation of the stem cells into the neuroectoderm, and in vitro, an improved differentiation was observed due to the elongation of embryoid bodies (EB). Specially designed scaffolds termed MatriFilamentSystems are used here as BME in the micrometer range. In vivo, the neural tube folds from the neural plate. Mechanical forces are used as well as apical constriction of the cells, which reduces the apical surface. A special extracellular matrix (ECM) for embedding of the EBs is used to provide mechanical impulses in vitro. Cryosectioning of cerebral organoids and subsequent immunofluorescence staining (DAPI blue, PAX6 green, TUJ1 red) reveals the spatial organization within the organoids. All images copyright TU‐Ilmenau.

In addition to mechanotransducive factors, spatial information plays an important role in organoid development. As described by Lancaster et al., the formation of a neuroectoderm and differentiation in the neuronal direction can be improved through the use of floating filaments, which serve as scaffolds for polarization.^[^ ^79^ ^]^ These filaments are micro‐scalic, therefore only a small fraction of cells is in direct contact with filaments (5–10%); nevertheless, they shape the whole embryoid body (EB). In addition to using microfilaments for the iPS cells, the shaping of the EBs could be further improved by an external meso‐scalic scaffold, which we introduced in the form of the MatriGrid carrier with oval cavities (750 µm × 300 µm × 300 µm). In such MatriGrids, the microfilaments are flushed into the cavities to facilitate the formation of EBs and subsequent organoid generation. The cells are seeded into the MatriGrids containing microfilaments, so‐called MatriFilamentSystem (**Figure** **2**). The desired and properly shaped EBs are formed in high quantities. Subsequently, they can be harvested for further processing or differentiation into corresponding organoids and used for a variety of applications (Figure 2).^[^ ^85^ ^]^ Thus, our in vitro organoid culture systems are suited for the growth of cerebral organoids until they reach a certain size and stage of development. Although our technique offers several advantages, further optimization steps need to be done to support the development of more natural brain organoids on our platform.

There are now many protocols to grow and develop organoids, whereby a rough distinction can be made between guided and unguided approaches. In guided approaches, individual brain regions can be modeled and fused together to form so‐called assembloids. This allows the interaction of specific brain areas to be studied by such assembloids.^[^ ^48^ ^]^ A drawback of commonly used brain organoids is the underrepresentation of non‐ectodermal‐originating cells like endothelial cells, which are necessary for the formation of the neurovascular system. In vivo, the brain's neuro‐vascularly coupled architecture forges two formidable, complex, far‐from‐equilibrium systems that, in tandem, regulate energy and matter, orchestrate information across spatiotemporal scales, and mediate cognition. Indeed, the cerebrovascular network formed by ≈100 billion vessels (equivalent to 400 miles) with complex geometries (e.g., fractal‐like), topology, and networks across spatial scales (i.e., vessel diameters ranging from µm to mm^[^ ^86^ ^]^ delivers vital molecules (e.g., oxygen, glucose) to and clears metabolic wastes (e.g., carbon dioxide, amyloid) from ≈200 billion brain cells (both neurons and non‐neurons).^[^ ^86^ ^]^ The absence of the vasculature in cerebral organoids in vitro restricts growth and therefore hinders cell maturation, as the diffusion of oxygen and nutrients is limited to ≈300 µm, and the organoids therefore form a necrotic core inside once they reach a certain size. This is one drawback of our current in vitro organoid culture technique using MatriFilamentSystems that needs to be addressed in future studies.

There are some methods to achieve vascularization, such as assembling brain and vascular organoids,^[^ ^87^ ^]^ adding endothelial cells in the step of EB formation or coculturing brain organoids with them, forcing endothelial differentiation by adding growth factors such as VEGF during differentiation, microfluidic brain‐on‐a‐chip approaches, or transplanting organoids into immuno‐diffuse mice. However, the perfusability and, thus, the modeling of the blood‐brain barrier in these systems remains a problem.^[^ ^83^ ^]^ The incorporation of the blood‐brain barrier would be crucial for proper drug testing.^[^ ^87^, ^88^ ^]^

Another underrepresented cell type in brain organoids is oligodendrocytes, which are important for the formation of white matter. White matter is crucial for inter‐ and intraregional communication in the human brain. While the cell bodies are located in the gray matter of the brain, the axons extend macroscopically within the white matter and form bundles and networks between spatially separated areas of the brain. The axons are covered with an insulating myelin layer that enables rapid signal transmission. An attempt to reproduce the white matter in vitro was made by Osaki et al.^[^ ^89^ ^]^ In this study, two organoids were placed a few mm apart from each other. A channel was placed between the two organoids to allow an axonal connection between them. The two organoids actually stretched their axons through the channel and formed an axon bundle with a diameter of 120 µm over a period of 8 weeks. To monitor the neuronal activity of the organoids, multielectrode arrays were inserted into the scaffolds.^[^ ^89^ ^]^ With the formation of an axonal connection, the burst activity between the connected organoids becomes synchronized. The performance of axonally connected organoids was compared with that of fused and simple organoids. Using the signal propagation speed, conclusions could be drawn that the signal propagation via axon bundles is significantly faster than in directly fused organoids. Furthermore, oscillatory activity is enhanced in this approach.^[^ ^89^ ^]^ Single‐cell RNA sequencing of the single, axonal connected and fused organoids provided indications that the neurons in the axonal connected organoids are more developed than in the others. Interestingly, it was also shown that optogenetic stimulation of the axon bundles between the organoids can trigger short‐term plasticity. When the stimuli were reapplied, a shortened induction time and a sustained increase in burst activity were observed.^[^ ^89^ ^]^

While this study of Osaki et al. focused on the intraregional communication of two organoids via axon bundles, it would be exciting to integrate further organoids and brain regions into the network and read out the communication and learning ability. An example of an assembloid comprising multiple organoids was given by the group of S. Pasća.^[^ ^90^ ^]^ They mimicked the complex human sensory pathway from the periphery to the brain by generating somatosensory organoids, dorsal spinal cord organoids, diencephalic (thalamic) organoids, and cerebral cortical organoids from pluripotent stem cells and then functionally assembling them to create four‐part human ascending somatosensory assembloids (hASAs). Measuring neural activity is an important criterion for analyzing the functionality of organoids and assembloids. For this purpose, stretchable, tissue‐mimetic mesh electrodes were developed by Le Floch et al.^[^ ^91^ ^]^ that can be integrated into the organoid and enable chronic monitoring of neuronal activity within the organoid over extended periods of development. Furthermore, Sheng et al.^[^ ^92^ ^]^ introduced a tissue‐level soft mesh microelectrode array that was integrated into the developing vertebrate brain during embryogenesis. A sub‐micrometer‐thin mesh was used, which was placed on the 2D neural plate and folded during neurulation. The dynamic process of morphological remodeling of the developing central nervous system was not measurably disturbed by the attachment of the electrodes, allowing reliable monitoring of the neural activity of the process.^[^ ^92^ ^]^ A promising future application for such systems would be their use as computing devices by means of neural automata^[^ ^16^, ^17^ ^]^ to which we could refer to as *orgamats*, i.e., organoid automats.^[^ ^14^, ^15^ ^]^ These systems currently still appear to be science fiction, but recent advances and novel techniques might enable them in the near future.

While artificial intelligence (AI) is all the rage these days with its large language models,^[^ ^93^ ^]^ the artificial neurons within the computers, their interconnection in networks, and the associated learning rules are far removed from the actual physiology of the brain. This is evident in the energetic efficiency of a human brain. Impressively, this complex system, which weighs ≈1.5 Kg, operates at only ≈20 watts, the energy of a light bulb.

**Figure 3:**
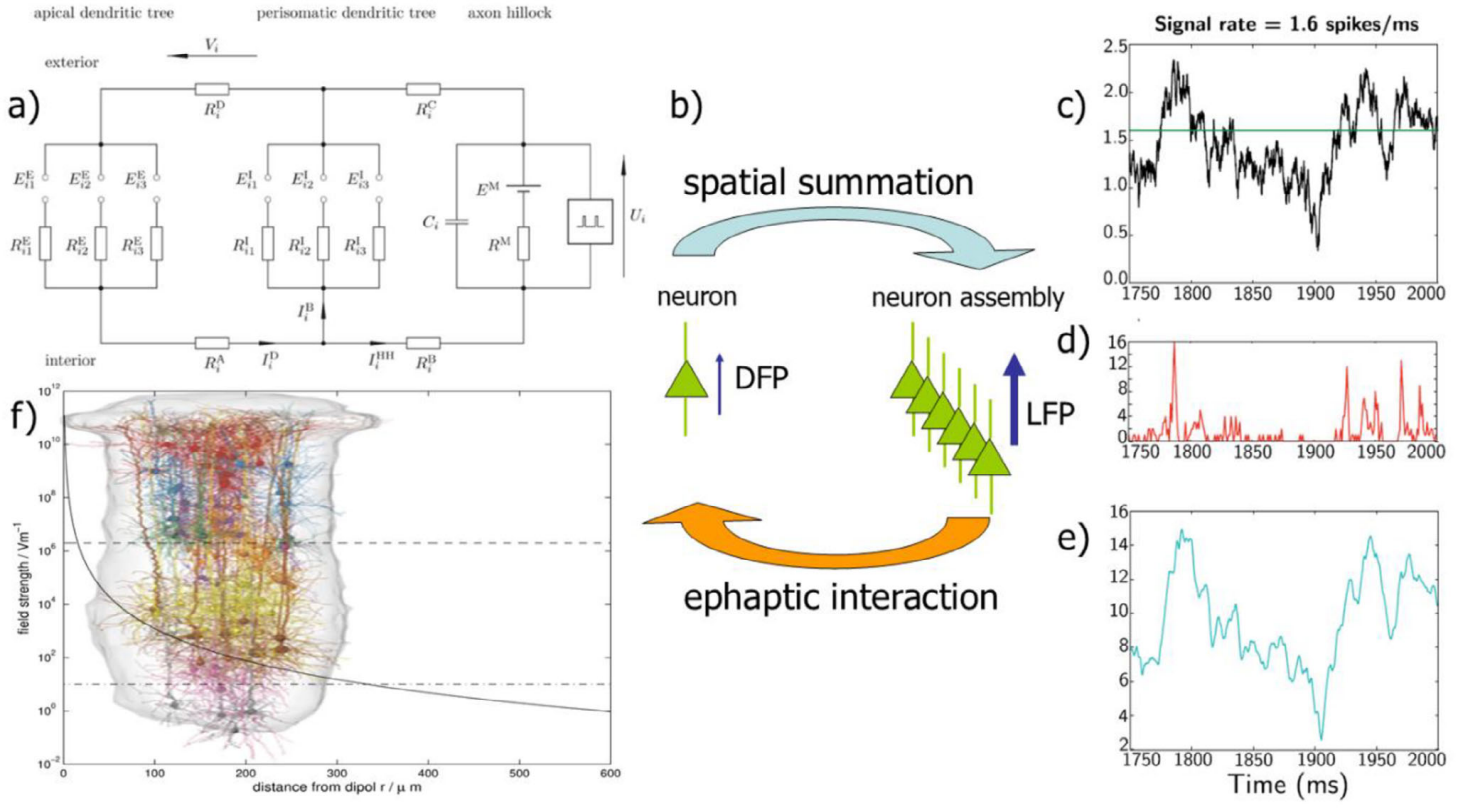
a) Electronic equivalent circuit for ion currents through three fundamental compartments of a cortical pyramidal cell and for the resulting electric dipole moment.^[^ ^6^ ^]^ [Figure 2] b) DFP of a pyramidal neuron and spatial summation to the columnar LFP with ephaptic feedback to individual neurons. c) Simulated thalamic input signal,^[^ ^6^ ^]^ [FIG. 3(B)] d) spike rate,^[^ ^6^ ^]^ [FIG. 3(K)] and e) resulting LFP.^[^ ^6^ ^]^ [FIG. 3(Q)] f) Cortical column^[^ ^12^ ^]^ [FIG. 21.6(a)] and theoretical range of the simulated ephaptic feedback.^[^ ^5^ ^]^ [FIG. 1.25.] All figures are reprinted with permission: (a), (c,d): Creative Commons Attribution License by beim Graben and Rodrigues, Frontiers in Computational Neuroscience (2013); (f) by Springer Nature.

Therefore, the use of brain organoids made from iPS cells as computing units is being seriously discussed in science and has already been implemented.^[^ ^94^ ^]^ The cortical column, or hypercolumn or functional column, is considered to be a collective of constituent functional elements in the cortex (cf. inset of **Figure** **3**f).^[^ ^8^, ^11^, ^12^, ^13^ ^]^ Synchronous activity of 10 000–100 000 neurons leads to local field potentials (LFP) and far‐field EEG signals according to simulations.^[^ ^8^, ^9^, ^10^, ^95^ ^]^ It is well‐known that the sources of electric field potentials (DFP, LFP, EEG) in the brain are the pyramidal neurons, situated mainly in layers III, V, and VI of the cortical columns, where their apical dendritic trunks form aligned palisades.^[^ ^6^, ^96^, ^97^, ^98^ ^]^ The apical dendritic tufts as well as the basal dendritic trees are covered with dendritic spines forming mainly excitatory synapses, while the perisomatic dendritic trees are equipped with mainly inhibitory synapses. In extracellular fluid, inhibitory synapses act as current sources, whereas excitatory synapses contribute current sinks. Because of their spatial separation along the apical dendritic trunks, pyramidal cells appear as electric dipoles that are surrounded by their characteristic DFP. Integrating these DFPs over a cortical column or hypercolumn entails the LFP, and its low‐pass filtered averages over entire brain regions give rise to the emergent EEG.^[^ ^6^, ^98^ ^]^

What is currently missing and what should be addressed by future studies is a research platform that allows the construction of sufficiently complex cerebral systems, e.g., cortical columns, hypercolumns, and assembloids thereof, and the investigation of their electrical properties despite the reduction of the size. It would be particularly desirable to be able to investigate synergistic properties of neuronal assemblies, such as dendritic field potentials (DFPs), local field potentials (LFPs), EEG, and ephaptic feedback effects of the summed field on individual cells.^[^ ^6^, ^99^, ^100^, ^101^ ^]^ In a series of studies, we have developed biophysical observation models for cortical field potentials.^[^ ^6^ ^]^ Starting with the dipole model of a single pyramidal cell, they suggested an electronic equivalent circuit, comprised of three compartments: one for the excitatory synapses at the apical dendritic tuft, one for the inhibitory synapses at the perisomatic dendritic tree, and another one for the axon hillock as a model of the spiking dynamics (Figure 3).^[^ ^6^, ^98^ ^]^ Further investigations and optimizations will be necessary to establish this system.

In order to test and to further improve such biophysical observation models, experimental organoid and assembloid prototypes would be mandatory. The current three‐compartmental model of a pyramidal cell is, e.g., not able to explain the well‐known polarity reversal of LFP when a micro‐electrode passes through different columnar layers. On the one hand, this could be straightforwardly remedied by spatially implemented neural networks with explicit layered structures, such as neural fields.^[^ ^7^ ^]^ On the other hand, an electronic four‐compartmental model of the single pyramidal cell could be devised by additionally taking the excitatory synapses at the basal dendritic tree into account.^[^ ^96^, ^97^ ^]^ Assembloid technology could substantially mitigate the problems of vascularization, myelination, and electrophysiological signal complexity by using divide‐and‐conquer‐solutions to avoid creating monolithic organoids, and rather implement multiple small and manageable organoid networks that communicate with each other via recent developments.^[^ ^102^ ^]^ In particular, feedback electrical fields would have two purposes: 1) To enable pluripotent stem cells to differentiate into different neurons via recent approaches;^[^ ^103^, ^104^ ^]^ 2) To enable neuronal signalling, communication, and computations. Intermediate steps could also employ optogenetics to further dissect neuronal dynamics and control it for computational purposes using recent approaches.^[^ ^105^ ^]^

Organoid and assembloid prototypes could be used for testing the hypothesis of ephaptic field effects, which states that neuronal dynamics could be directly modulated by their coupling to the surrounding electromagnetic fields (Figure 3).^[^ ^5^, ^99^, ^100^, ^101^ ^]^ The extracellular field strength exhibits an exponential decay. The horizontal lines indicate the required strength for triggering action potentials (upper line) and for the significant causation of physiological effects (lower line).^[^ ^5^ ^]^ Interestingly, the spatial range of the latter almost coincides with the diameter of the cortical column, indicated by the inset of Figure 3f.^[^ ^11^, ^12^ ^]^

Moreover, the tentative exploitation of brain organoids and assembloids as future computing devices as *orgamats* requires a better understanding of the coupling between neuronal dynamics and the electromagnetic fields in extracellular space, and more experimental data validating our hypothesis. In the classical animate paradigm, DFP and LFP are recorded in 2D neuronal cultures, analyzed and subsequently classified by an external computer, and then fed back as electrical stimulation to the neuronal population in a closed‐loop arrangement. Using AI and reinforcement learning then allows the training of the culture by means of Hebbian correlation learning to accomplish particular tasks, e.g., controlling a simple robot.^[^ ^14^, ^15^ ^]^ A promising programming interface for the training of *orgamats* was recently able to develop neural automata or neural Turing machines.^[^ ^16^ ^]^

**Figure 4:**
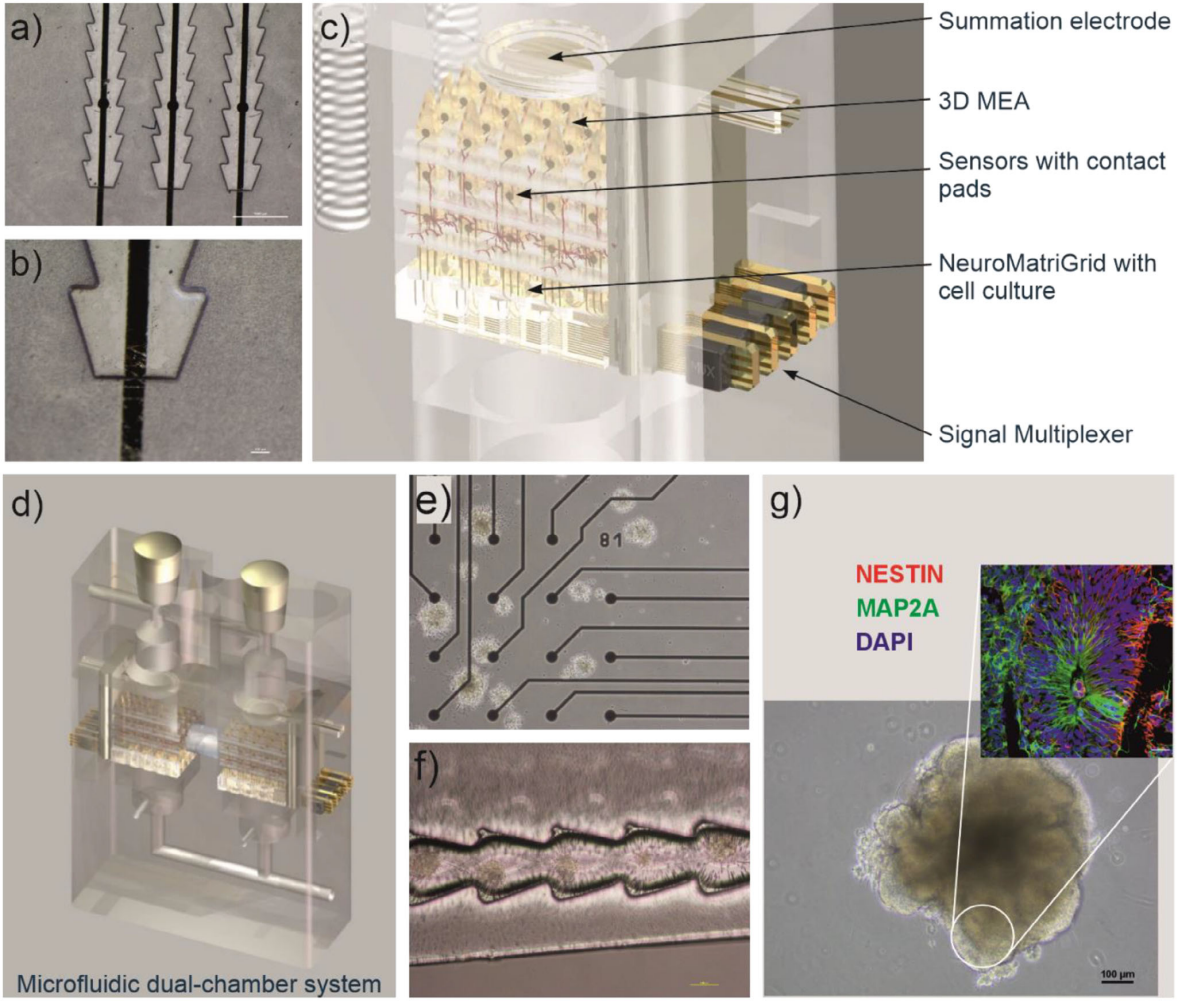
a,b) Plastic films with integrated gold electrodes. c) Bioreactors with assembled NeuroGrids with integrated electrodes. d) Microfluidic dual‐chamber systems for studying the interaction of two cell assemblies. e) Brain organoids on an electrode array. f) Assembly of brain spheroids. g) Differentiating brain organoid. All images copyright TU‐Ilmenau.

Brain organoids that represent different functional areas of the brain could be assembled over distance, as mentioned before.^[^ ^98^ ^]^ With the MatriGrid Technology, we have a tool to combine different organoids in microfluidic substrates (**Figure** **4**).^[^ ^106^ ^]^ Microfluidic coupling allows the study of self‐organization by varying the possible introduction between two cell populations. (Figure 4). Different cell types (functional or species) may be combined and coupled by microfluidic means, as shown in Kanagasabapathi et al. and Osaki et al.^[^ ^16^, ^89^, ^107^, ^108^, ^109^ ^]^ After establishing and optimizing our in vitro organoid culture platform, we will investigate these topics in future experiments.

Our goal will be to establish a research platform for the integration of different neuronal cell assemblies up to brain organoids, which should achieve the following: directed growth of neuron palisades on plastic supports with integrated electrodes, assembly of brain organoids, integration of plastic supports with neuron bundles in ceramic‐based electrode structures, and measurement of pointwise (DFP) and sum potentials (LFP, EEG).

Specific questions can be investigated using specific setups at different scales, be it signal propagation along nerve axons, the precise investigation of learning behavior at the synaptic cleft, the transition from individual neuronal activity to aggregate activity and its possible feedback on the behavior of individual neurons, the hierarchical organization of entire neuronal processing systems such as the brain, or even the possible formation and evolution of complex structures due to the interaction of competing or cooperating cell assemblies.

## 4 Resume: Technical Hybrid Systems are Well Suited as an Investigation Platform for Biological Systems

Progress in cell cultivation technologies like micro bioreactor technology, stem cell culture may lead to sufficiently complex, segregated oligocellular systems, able to answer questions of experimental biology, pharmaceutical science, and medicine.

The task is now to find a suitable bio‐technical hybrid‐ or model‐system for the right description level of the scientific or technical problem. With such systems, one could examine the conditions of self‐organization and production of information in biological systems!

By this, we can add to the modern molecular biology/synthetic biology approach, based on biological, chemical, and biochemical tools, a complementary approach by combining technical systems with methods of molecular biology.

## Conflict of Interest

The authors declare no conflict of interest.
